# Adult Separation Anxiety and TCI-R Personality Dimensions in Patients with Anxiety, Alcohol Use, and Gambling: A Preliminary Report

**DOI:** 10.1155/2014/680985

**Published:** 2014-07-03

**Authors:** Gino Pozzi, Angelo Bruschi, Andrea De Angelis, Marco Pascucci, Daniele Stavros Hatzigiakoumis, Paolo Grandinetti, Marco Di Nicola, Stefano Pini, Luigi Janiri

**Affiliations:** ^1^Institute of Psychiatry and Psychology, Catholic University of the Sacred Heart, Rome, Italy; ^2^Department of Clinical and Experimental Medicine, Section of Psychiatry, University of Pisa, Pisa, Italy

## Abstract

*Background*. Nowadays, adult separation anxiety disorder (ASAD) is an established diagnostic category but is little investigated in subjects with addictive behaviours. *Objective*. To assess the presence of ASAD among patients with addictive disorders in comparison with anxiety patients and measure the personality correlates in all these groups. *Methods*. 103 outpatients, meeting DSM-IV-TR criteria for anxiety disorders (38 patients), alcohol dependence (30 patients), or pathological gambling (35 patients), were assessed by the Structured Clinical Interview for Separation Anxiety Symptoms (SCI-SAS) and the Adult Separation Anxiety Checklist (ASA-27) for separation anxiety and by the Temperament and Character Inventory-Revised (TCI-R) for personality characteristics. *Results*. ASAD is detected in 34.2% of anxiety patients, 13.3% of alcoholics, and 11.4% of gamblers. Separation anxiety scores correlate positively with harm avoidance and negatively with self-directedness in all groups; further correlations are seen among addictive patients only, that is, self-transcendence for gamblers and cooperativeness for both alcoholics and gamblers. *Conclusions*. The prevalence of ASAD is lower among addictive patients than in those with anxiety disorders; correlations are found between separation anxiety and specific TCI-R dimensions, with some matching across the three diagnostic groups.

## 1. Introduction

Separation anxiety can be defined as a condition burdened by an excessive and inappropriate display of fear and distress when the individual is faced with situations of separation from home or from a specific attachment figure. As is well known, the discomfort arisen by the separation from an attachment figure is related to the ordinary childhood development [1, 2], with a probable evolutionary purpose: the retention of the human offspring, still inept, near its main caregiver [3].

Only if the sensitivity to the separation becomes excessive and prolonged, with intense anxiety and interference in daily life activities or normal development, it can be diagnosed as separation anxiety disorder (SAD).

Although this disorder has been classically defined as a childhood phenomenon, growing evidence exists that separation anxiety may have an adult onset (adult separation anxiety disorder, ASAD), regardless of history of childhood separation anxiety disorder (CSAD) [4].

Many studies have been conducted to evaluate the clinical and epidemiological characteristics of this disease [5, 6], the prevalence and gender differences [7], the impairment of functioning [5], the correlation with specific biomarkers [8, 9], and the temperament and character dimensions [10].

Apart from general population studies [5], the ASAD comorbidity has been investigated mainly in patients with mood disorders [11], anxiety disorders [7], posttraumatic stress disorder [12], and personality disorders [4, 7].

Recently, the American Psychiatric Association, in its DSM-5, decided to create a brand new specific ASAD category within the general section of the anxiety disorders [13].

Apart of the seminal study of Loas et al. [14], to the best of our knowledge, no clinical study has further investigated the presence of ASAD among patients with addictive disorders, with particular reference to gambling.

The objective of this study is to assess the presence of adult separation anxiety in patients with chemical or behavioral addictions, in comparison with a clinical sample of anxiety patients, and to measure the personality correlates in all the groups using the Temperament and Character Inventory-Revised (TCI-R) [15].

## 2. Materials and Methods

### 2.1. Participants

Subjects were recruited, during the year 2012 and in a consecutive manner, among clients referring to the adult psychiatric outpatient clinic of the “A. Gemelli” University General Hospital in Rome.

Inclusion criteria were (1) currently meeting DSM- IV-TR [16] criteria for anxiety disorder (38 patients), alcohol dependence (30 patients), or pathological gambling (35 patients); (2) having an age of 18 to 65 years; (3) for the anxious patients, spending at least one month of integrated treatments including benzodiazepines and/or selective serotonin reuptake inhibitors-serotonin norepinephrine reuptake inhibitors (SSRI-SNRI); (4) for the alcoholics and gamblers, spending at least one month of a specific rehabilitation program requiring total abstinence from the addictive behavior (alcohol abuse or pathological gambling) and the possibility, upon the clinician's advice, of a maintenance treatment with mood stabilizers (valproate, gabapentin, and pregabalin).

Subjects were excluded if any of the following conditions were present: (1) a diagnosis of mental retardation or documented IQ < 70; (2) any other current axis I DSM-IV-TR diagnosis; (3) unstable general medical conditions; (4) clinically significant prestudy physical exam, electrocardiogram, laboratory, or urinalysis abnormalities indicating serious medical disease impairing evaluation; (5) pregnant or breast-feeding women; (6) recent use of not prescribed drugs.

### 2.2. Procedures

DSM-IV-TR current diagnosis was preliminarily established by trained psychiatrists (G. P. and A. B.). Then an anamnestic interview was administered in order to obtain sociodemographic information and psychiatric history.

All participants were interviewed by specifically trained interviewers (M. P., P. G., and A. D. A.) using the Structured Clinical Interview for Separation Anxiety Symptoms (SCI-SAS) [17] and the Adult Separation Anxiety Checklist (ASA-27) [18].

Moreover they were administered the TCI-R, the Italian version [19, 20].

The study was conducted in accordance with the latest revision of the Declaration of Helsinki and the rules of Good Clinical Practice (ICH-GCP): all subjects provided written informed consent after a complete description of the study procedures and participated without receiving any form of payment. In order to ensure anonymity all the acquired data were deidentified before any further manipulation, ensuring an adequate level of protection, using a double level of data encryption.

### 2.3. Separation Anxiety Assessment

The categorical assessment of CSAD and ASAD was conducted using the SCI-SAS [17]. This semistructured interview contains items derived from the DSM-IV-TR criteria for CSAD with symptoms modified for adulthood. According to the DSM-IV, endorsement of three or more of the eight criterion symptoms was used as a threshold to determine a diagnosis of CSAD and of ASAD. For the diagnosis were required a symptom duration of at least 4 weeks and clinically significant distress or impairment in social, academic, occupational, or other important areas of functioning.

The dimensional measure of ASAD was performed by administering a self-report questionnaire, the ASA-27 [18]. The ASA-27 is a 27-item inventory which rates symptoms of adult separation anxiety after the age of 18, having high levels of internal consistency (Cronbach's *α* = 0.89) and test-retest reliability (*r* = 0.86; *P* < 0.001); moreover it has shown concurrent validity with clinical assessments of ASAD, with a cut-off score of twenty-two.

### 2.4. Personality Assessment

Personality was investigated by means of the TCI-R [20]. This is a 240-item, five-point Likert scale, a reliable and valid questionnaire that measures seven dimensions of personality: four dimensions of temperament (i.e. harm avoidance (HA), novelty seeking (NS), reward dependence (RD), and persistence (P)) and three character traits (i.e. self-directedness (SD), cooperativeness (CO), and self-transcendence (ST)) [15]. Internal consistency of the different personality dimensions in the Italian adaptation ranged between *α* = 0.78 and *α* = 0.89 [8].

### 2.5. Statistical Analysis

Statistical analysis was conducted using SPSS for Windows, Version 15 (SPSS Inc., Chicago, Illinois). Dichotomous data were compared by chi-square test using the Fisher or the Yates corrections as appropriate. Continuous data were expressed as means ± standard deviation and compared by one-way ANOVA. The principal outcome analysis consisted of nonparametric Kruskal-Wallis H test for comparison between the three groups. Spearman's rank correlation coefficient was employed to examine the relationship between continuous variables. All tests were 2-tailed, with statistical significance set at *P* < 0.05.

## 3. Results

The study group included 38 patients with anxiety disorders (mostly generalized anxiety disorder and panic disorder), 30 patients with an alcohol use disorder (mostly alcohol dependence), and 35 patients with a gambling disorder (i.e., pathological gambling). The demographic characteristics are summarized in Table 1.

*Separation Anxiety in the Three Study Groups. *No statistically significant difference was found in the frequency of CSAD across the three diagnostic groups (*P* = 0.227). As a category, ASAD is detected in about one-third of the anxiety patients in comparison to some ten percent in the other study groups (*P* < 0.05). If separation anxiety is assessed dimensionally, mean values do not differ in the three groups (*P* = 0.777). Finally, when the cut-off of ASA-27 is taken into account, the number of pathological gamblers scoring above the threshold is close to the amount found in the anxiety group (Table 2).

*Correlation of Separation Anxiety Scores and Personality Dimensions. *Spearman's rho correlation coefficients of the seven TCI-R main dimensions with the ASA-27 rough scores are shown in Table 3. A strong positive correlation is found between the HA and the separation anxiety symptom scores: this is statistically significant in all the three groups with a maximum in the anxiety (*P* < 0.01). Another strong correlation, albeit inverse, is found in all three groups between the SD and the ASA-27 scores, with maximum statistical significance for pathological gambling and anxiety patients (*P* < 0.01). Further correlations are seen among addictive patients only, that is, ST for gamblers (*P* < 0.01) and CO for both alcoholics (*P* < 0.05) and gamblers (*P* < 0.05).

## 4. Discussion

With reference to study aims, the assessment of separation anxiety in the three groups showed differences in the categorical prevalence of ASAD, which was lower among alcoholics and gamblers; moreover, the scores of separation anxiety showed specific correlations with some TCI-R dimensions.

### 4.1. Prevalence Rates

Our data almost confirm the previous literature results. Considering the prevalence of separation anxiety in general population, the National Comorbidity Survey Replication (NCS-R) [6] showed a 12-month ASAD prevalence of 1.9% and a lifetime prevalence of 6.6% [5]; more than half of those diagnosed with ASAD had a history of mood disorders (53%), and the majority (75%) had received or were in treatment for emotional problems. Scanning the clinical studies, Pini et al. [21] reported that 42.4% of the anxiety and mood disorder outpatients screened also met the ASAD criteria. The prevalence of ASAD in dependent personality disorder patients was examined in a large patient sample with alcohol or drug addiction compared to nonpatient controls [14]: the rates in the control participants were from 2 to 5%, whilst in patients the results were significantly higher, ranging from 6 to 31%; in both cases, those with alcohol addictions had the lowest prevalence of ASAD. As reported, we found that the ASAD lifetime frequency rate is 11.4% in the gambling, 13.3% in the alcohol, and 34.2% in the anxiety sample. To the best of our knowledge, this is the first study assessing the frequency of ASAD among gamblers, so confirming that the cooccurrence of separation anxiety and addictive disorders is clearly less frequent than the cooccurrence of separation anxiety with mood or anxiety disorders.

### 4.2. TCI-R Measures

Many studies showed a correlation between TCI-R dimensions and anxiety disorders, gambling and alcohol addiction.

Regarding anxiety, all studies agreed on two core points: a high correlation between the temperamental dimension of harm avoidance (HA) and anxiety symptoms and an important inverse correlation between the character dimensions of self-directedness (SD) and anxiety symptoms, with HA scores increasingly higher and SD increasingly lower with the illness severity growing [22–25]. However Lu et al. [26] suggested that, although anxiety is linked to high HA scores, only high novelty seeking (NS) appears as a good predictor of anxiety; indeed in agreement with the original viewpoint by Cloninger, people with high NS can show anxiety characterized by generalized turmoil or alarm without specific premonitory cues, frequently bodily pains, and slow fatigability [27]. According to these results HA and NS would then be connected and could be the litmus paper of two different forms of anxiety.

Regarding addictive disorders, alcohol-dependent patients in general scored higher on NS and lower on SD than controls: according to the authors [28, 29] the lower SD indicates a predisposing factor for alcohol dependence, even if it could be seen as either preceding or consequent upon alcohol pathological use. Instead, pathological gamblers showed higher NS values, lower SD, and lower cooperativeness (CO) with higher NS associated with earlier age of onset of problem gambling [30–32].

The only study that measured the TCI-R dimension in ASAD reported an elevation in HA, reward dependence (RD), and self-transcendence (ST) levels and lower SD scores, with ASAD patients showing quantitatively greater severity in high HA and lower SD; the TCI-R profile of these subjects seems very similar to patients with anxiety disorders [10].

The results of our research partially confirm all these literature findings, even if there are some peculiar differences that deserve some clarification. The HA strongly correlates with separation anxiety symptoms in all the three groups: the score of this dimension indicates fear of the unknown and shyness with strangers, which could lead to avoidance behaviour especially in new situations. High levels of HA are linked to overcaution, insecurity, and passivity [15, 33]. On the contrary, NS does not correlate with separation anxiety in any of the groups, confirming that this kind of anxiety is different than that of the other anxiety disorders, as mentioned above.

Focusing on the other results, SD shows a strong inverse correlation with ASA-27 scores in all the three clinical samples. Since SD could be defined as the measure of resourcefulness and self-acceptance [34], low levels of SD are linked to irresponsibility, inefficiency, weakness, and bad self-reliance. This is consistent with a fundamental role of separation anxiety in the integration of functions of the self.

Cooperativeness seems to be inverse-correlated with ASAD symptoms only within the addictive sample, both in alcohol and gambling. Considering that CO is the capacity to understand and accept other people [15], low levels of CO are linked to intolerance, incomprehension, nonsociability, and indifference.

The ST dimension appears to be characteristic of the gamblers. ST refers to magical thinking, unselfishness, and superstition, prototypical features of a behavioural addiction [32].

## 5. Conclusions

This study is the first one assessing the frequency rates of ASAD in both chemical and behavioural addiction, as compared to a sample of anxiety patients. In addition we pointed out the temperament and character correlations of separation anxiety in these patients, which were shared across the disorders (HA, SD) or typical of the addictions (CO, ST). Limitations of this preliminary investigation include a small sample size, some imbalance in the demographic characteristics of the three populations, and a lack of clinical subtyping. So, our observations need to be replicated in larger groups, also widening the target on other chemical and behavioural addictions and taking into account further comorbidities.

## Figures and Tables

**Table 1:** Demographic characteristics.

Characteristics of patients	ANX (*N* = 38)	ALC (*N* = 30)	PG (*N* = 35)
Age (mean ± SD)	39.82 ± 11.97	51.03 ± 8.68	50.63 ± 13.07
Gender			
Male	8 (21.1%)	13 (43.3%)	28 (80.0%)
Female	30 (78.9%)	17 (56.7%)	7 (20.0%)
Marital status			
Single	20 (52.6%)	15 (50.0%)	9 (25.7%)
Married	16 (42.1%)	11 (36.7%)	18 (51.4%)
Divorced/widowed	2 (5.3%)	4 (13.3%)	8 (22.9%)
Education			
Middle school	7 (18.4%)	7 (23.3%)	12 (34.3%)
High school	22 (57.9%)	15 (50.0%)	18 (51.4%)
University degree	9 (23.7%)	8 (26.7%)	5 (14.3%)
Occupation			
Employed	21 (55.3%)	13 (43.3%)	20 (57.1%)
Unemployed	3 (7.9%)	13 (43.3%)	3 (8.6%)
Other	14 (36.8%)	4 (13.3%)	12 (34.3%)

ANX: anxiety disorders; ALC: alcohol use disorders; PG: pathological gamblers.

Other: student, retired, and housewife.

**Table 2:** Separation anxiety across the three study groups.

	ANX (*N* = 38)	ALC (*N* = 30)	PG (*N* = 35)	*P* value
CSAD	8 (21.1%)	3 (10.0%)	2 (5.7%)	0.227
ASAD	13 (34.2%)	4 (13.3%)	4 (11.4%)	0.028
ASA-27 (mean value ± SD)	23.45 ± 16.75	22.50 ± 12.92	20.66 ± 12.78	0.777
ASA-27 (score ≥ 22)	17 (44.7%)	9 (30.0%)	15 (42.8%)	0.422

ANX: anxiety disorders; ALC: alcohol use disorders; PG: pathological gamblers.

CSAD: childhood separation anxiety disorder; ASAD: adult separation anxiety disorder.

**Table 3:** Correlation of ASA-27 scores with TCI-R dimensions across the three study groups (Spearman's rho).

	ANX	ALC	PG
Novelty seeking	−0.160	−0.097	−0.060
Harm avoidance	0.670∗∗	0.384∗	0.383∗
Reward dependence	0.240	−0.156	0.003
Persistence	0.192	0.114	0.242
Cooperativeness	0.093	−0.395∗	−0.361∗
Self-directedness	−0.482∗∗	−0.386∗	−0.566∗∗
Self-transcendence	0.144	0.088	0.436∗∗

ANX: anxiety disorders; ALC: alcohol use disorders; PG: pathological gamblers.

*P < 0.05; ∗∗*P* < 0.01.
